# Development of a micromanipulation method for single cell isolation of prokaryotes and its application in food safety

**DOI:** 10.1371/journal.pone.0198208

**Published:** 2018-05-31

**Authors:** Marisa Hohnadel, Myriam Maumy, Renaud Chollet

**Affiliations:** 1 Biomonitoring R&D, Life Science Applied Solutions, Merck, Molsheim, France; 2 Institut de Recherche Mathématique Avancée, UMR7501, Université de Strasbourg, Strasbourg, France; The Ohio State University, UNITED STATES

## Abstract

For nearly a century, conventional microbiological methods have been standard practice for detecting and identifying pathogens in food. Nevertheless, the microbiological safety of food has improved and various rapid methods have been developed to overcome the limitations of conventional methods. Alternative methods are expected to detect low cell numbers, since the presence in food of even a single cell of a pathogenic organism may be infectious. With respect to low population levels, the performance of a detection method is assessed by producing serial dilutions of a pure bacterial suspension to inoculate representative food matrices with highly diluted bacterial cells (fewer than 10 CFU/ml). The accuracy of data obtained by multiple dilution techniques is not certain and does not exclude some colonies arising from clumps of cells. Micromanipulation techniques to capture and isolate single cells from environmental samples were introduced more than 40 years ago. The main limitation of the current micromanipulation technique is still the low recovery rate for the growth of a single cell in culture medium. In this study, we describe a new single cell isolation method and demonstrate that it can be used successfully to grow various types of microorganism from picked individual cells. Tests with Gram-positive and Gram-negative organisms, including cocci, rods, aerobes, anaerobes, yeasts and molds showed growth recovery rates from 60% to 100% after micromanipulation. We also highlight the use of our method to evaluate and challenge the detection limits of standard detection methods in food samples contaminated by a single cell of *Salmonella enterica*.

## Introduction

Diseases caused by foodborne pathogens have long been a serious threat to public health and food safety and remain a major concern to society. According to reports from the Centers for Disease Control and Prevention (CDC), approximately 48 million people in the United States become ill, 128,000 people are hospitalized, and 3000 people die from foodborne diseases each year [1, 2, 3].

Pathogens that cause foodborne diseases include bacteria, viruses, fungi, and parasites [3]. Thirty-one foodborne pathogens are identified in the United States: it is thought that viruses are the main cause of illness, while bacteria are the main cause of admission to hospital and death [4]. The common pathogens responsible for most outbreaks of foodborne disease are *Listeria monocytogenes*, *Escherichia coli* O157:H7, *Staphylococcus aureus*, *Salmonella enterica*, *Bacillus cereus*, *Vibrio* spp., *Campylobacter jejuni*, *Clostridium perfringens*, and Shiga toxin-producing *Escherichia coli* (STEC) [1, 3, 4].

Monitoring is the first step in the prevention of disease caused by foodborne pathogens. Effective inspection and detection methods are necessary to control pathogens in food products. For nearly a century, conventional microbiological methods have been standard practice for detecting and identifying pathogens in food. These methods continue to be reliable means of ensuring food safety. Nevertheless, driven by public demand in response to disease outbreaks, the microbiological safety of food has improved and various rapid methods, such as nucleic acid-based testing (e.g. qPCR), lateral-flow immunoassays, flow cytometry and biosensors, have been developed to overcome the limitations of conventional methods.

Ideally, a rapid test method should detect cell counts as low as one cell. According to the regulations for many food samples, the mandatory detection limit is less than 1 cell per 25 g or more of food [5, 6, 7]. Validation studies of assays for the rapid detection of foodborne pathogens generally evaluate the limit of detection (LOD) following inoculation of low levels of specific pathogenic strains. With respect to low population levels, the performance of a detection method is assessed by producing serial dilutions of a pure bacterial suspension to inoculate representative food matrices with highly diluted bacterial cells (≤ 10 CFU/ml). The accuracy of data obtained by multiple dilution techniques is not certain with respect to the Poisson distribution of low inoculation levels of bacterial cells [8]. Moreover, the dilution technique does not exclude some colonies arising from clumps of cells [9].

Micromanipulation techniques to capture and isolate single cells from environmental samples were introduced more than 40 years ago [10]. Most of the studies consisted of isolating single microbial cells from mixed populations under visual control to obtain pure cultures and investigate the ecology of microbial strains from natural habitats. The main obstacle of early systems was the low magnification, which was not sufficient for single bacterial cell micromanipulation [11]. With the continuous improvement of microscopes and the development of microcapillaries, axenic cultures have been successfully isolated from laboratory cultures and natural environments, demonstrating that cells captured by a micromanipulator can indeed be grown. Nevertheless, the main limitation of current micromanipulation is still the low recovery rate of single cell growth in culture medium [12], preventing the widespread use of this technique in laboratories [11].

In this article, we describe a new single cell isolation method to overcome this limitation and demonstrate that it can be used successfully to grow various types of microorganism from picked individual cells. We also highlight its use to evaluate the detection limit of standard pathogen detection methods in food samples contaminated by a single cell of *Salmonella enterica*.

## Materials & methods

### Microorganism cultures

All microorganisms were obtained from the American Type Culture Collection (ATCC), the Deutsche Sammlung von Mikroorganismen und Zellkulturen (DSMZ) or the National Collection of Type Cultures (NCTC) and were stocked in calibrated cryotubes at -80°C after growing in broth. We used the following strains: *Enterococcus faecalis* ATCC 19433, *Staphylococcus aureus* DSMZ 799, *Kocuria rhizophila* ATCC 9341, *Bacillus subtilis* DSMZ 347, *Bacillus cereus* ATCC 14579, *Bacteroides vulgatus* ATCC 8482, *Clostridium sporogenes* ATCC 19404, *Pseudomonas aeruginosa* DSMZ 1128, *Salmonella enterica subsp*. *enterica* NCTC 6676, *Escherichia coli* DSMZ 1576, *Proteus mirabilis* ATCC 29906, *Candida albicans* DSMZ 1386 and *Saccharomyces cerevisiae* ATCC 7754.

For the micromanipulation of bacterial cells, microorganisms were plated on agar media after thawing the cryotube at room temperature. Aerobic bacteria were plated on TSA (Merck ref. 146004) and incubated 24h at 32.5°C; anaerobic bacteria were plated on Columbia blood agar (COS) (Merck ref. 146559) and incubated under anaerobic conditions (Genbox, Biomérieux ref. 96124) 24h to 48h at 32.5°C; and yeasts were plated on Sabouraud dextrose agar (SDA) (Merck ref. 146028) and incubated 48h to 72h at 22.5°C.

### Development of the micromanipulation protocol for single bacterial cell isolation

To isolate microbial cells, we used a micromanipulator (Eppendorf TransferMan 4R, ref. 5193.000.012) equipped with a microinjector (Eppendorf CellTram Vario ref. 5176.000.017) mounted onto an inverse phase contrast microscope (Zeiss Axio Vert A1) at a magnification of 400x. The micromanipulator was used according to the manufacturer’s instructions.

#### Assessment of containers for micromanipulation

For the observation and handling of microbial cells with the micromanipulator, we evaluated five containers: a glass slide (RS France, ref. 29.201.311), a six-well plate (Corning Incorporated, ref. 3516), and Petri dishes of 35 mm diameter (Greiner BioOne, ref. 627102), 60 mm diameter (Greiner BioOne, ref. 628161) and 94 mm diameter (Greiner BioOne, ref. 633179).

The test organism was *Escherichia coli*. Directly after thawing the cryotube at room temperature, ten-fold serial dilutions in peptone salt (Fisher Scientific, ref. 1204.0487) were prepared. Approximately 10^5^ CFU were loaded on to each of the test bases: in a volume of 5 μl for the glass slide, 3 ml for the six-well plate, and 3 ml, 6 ml, and 18 ml respectively for the three Petri dishes.

#### Selection of capillary

Three types of glass capillary were evaluated for the purpose: IMSI/TEST capillary (Eppendorf, ref. 5175.117.000), Polar Body Biopsy FCH capillary (Eppendorf, ref. 5175.230.000) and TransferTip (ES) capillary (Eppendorf, ref. 5175.107.004).

The test organism was *Escherichia coli*. Directly after thawing the cryotube at room temperature, ten-fold serial dilutions in peptone salt (Fisher Scientific, ref. 1204.0487) were prepared. Each type of capillary was used to transfer 6 ml from the last dilution tube (containing approximately 10^5^ CFU/ml) into a 60 mm Petri dish, prior to microscopy.

#### Single cell culture methods

Two culture methods were evaluated for the growth of *E*.*coli*, *B*.*cereus*, *S*.*aureus*, *B*.*subtilis*, *P*.*aeruginosa*, *B*.*vulgatu*s and *C*.*albicans* after the isolation of single cells with the micromanipulator.

For each microorganism, one colony was picked from a 24h plate and resuspended in 9 ml of physiological saline. Ten-fold serial dilutions were prepared in physiological saline, and 6 ml transferred from the last dilution tube into an empty 60 mm Petri dish. Under the microscope, the tip of the capillary was moved close to a single cell of the test organism and the cell sucked into the capillary using the microinjector.

For the first culture method, the cell was released into 500 μl of physiological saline placed in an empty 94 mm Petri dish. Then 20 ml of agar medium were added to the plate following the standard pour plate method, using TSA (Biomérieux, ref. 41466) for aerobic bacteria, Schaedler (Thermo Scientific, ref. CM0437) for anaerobic bacteria and SDA (Merck, ref. 1.07315.0500) for the yeast. After cooling, the plates were incubated 24h to 48h at 32.5°C for bacteria and 48h to 72h at 22.5°C for yeasts. Ten replicates were performed per strain.

For the second culture method, single aerobic and anaerobic bacterial cells were deposited in one 5 μl drop of physiological saline on TSA and COS, respectively, and incubated 24h to 48h at 32.5°C. Single yeast cells were deposited in a 5 μl drop of physiological saline on SDA and incubated 48h to 72h at 22.5°C. Ten replicates were performed per strain.

#### Determination of the diluent

We tested four diluents for use during the micromanipulation: Tryptic soy broth (Merck, ref. STBMTSB12) half-diluted with sterile water; physiological saline (B.Braun, ref. 0066570E); peptone salt (Fischer Scientific, ref. 1204.0487); and a homemade Reasoner’s 2 broth (R2B).

The test organisms were *S*.*aureus* and *B*.*subtilis*. After thawing the cryotube at room temperature, the bacteria were plated on TSA and incubated 24h at 32.5°C, as described previously. One colony of each stain was then picked and resuspended in 9 ml of the test diluent. Ten-fold serial dilutions were prepared with the corresponding diluent and 6 ml from the last dilution tube (containing approximately 10^5^ CFU/ml) transferred into a 60 mm Petri dish prior to microscopy. Under the microscope, the tip of the capillary was moved close to a single cell of the test organism and the cell sucked into the capillary using the microinjector. Finally, the single bacterial cells were released in a 5 μl drop of physiological saline on TSA and incubated 24h to 48h at 32.5°C.

### Impact of physiological state on single cell viability after micromanipulation

We tested three physiological states of *E*.*coli* and *K*.*rhizophila*: frozen cells thawed at room temperature for 10 min and diluted in physiological saline; cells taken from a 24h culture plate; and cells in the exponential phase taken from a fresh liquid culture in TSB (Merck, ref. STBMTSB12) at 32.5°C under shaking. Optical density (OD) was measured using a biophotometer (Eppendorf, ref. 6131). When the exponential growth phase was reached, 1 ml of culture broth was diluted in 9 ml of physiological saline and ten-fold serial dilutions were prepared.

For each of the three starting conditions, 6 ml from the last dilution tube (containing approximately 10^5^ CFU/ml) were transferred into a 60 mm Petri dish. Under the microscope, the tip of the capillary was moved close to a single cell of *E*.*coli* or *K*.*rhizophila* and the cell sucked into the capillary using the microinjector. The single cells of *E*.*coli* and *K*.*rhizophila* were deposited in a 5 μl drop of physiological saline on TSA and incubated 24h to 48h at 32.5°C. Ten replicates were performed for each starting condition.

### Growth rate after micromanipulation

#### Determination of the growth rate

The growth rate of one single cell on culture medium after micromanipulation was evaluated on 13 strains, including aerobic bacteria, anaerobic bacteria, cocci, rods and yeasts: *E*.*coli*, *P*.*mirabilis*, *S*.*enterica subsp enterica*, *P*.*aeruginosa*, *B*.*cereus*, *B*.*subtilis*, *E*.*faecalis*, *S*.*aureus*, *K*.*rhizophila*, *B*.*vulgatus*, *C*.*sporogenes*, *S*.*cerevisiae* and *C*.*albicans*.

As described previously, one colony of each microorganism was picked from a 24h plate and resuspended in 9 ml of physiological saline. Ten-fold serial dilutions in physiological saline were prepared and 6 ml transferred from the last dilution tube into a 60 mm Petri dish. Under the microscope, the tip of the capillary was moved close to a single cell of the test organism and one cell sucked into the capillary using the microinjector. Single aerobic or anaerobic bacterial cells were deposited in a 5 μl or 10 μl drop of physiological saline on TSA or COS, respectively, and incubated 24h to 48h at 32.5°C. Single yeast cells were deposited in a 5 μl drop of physiological saline on SDA and incubated 48h to 72h at 22.5°C. Ten replicates were performed per strain.

#### Statistical analysis

The growth results from the 13 tested bacteria were statistically analyzed using proportional tests. At first, an exact proportional test (Fisher exact test) was applied to the dataset to compare the growth after micromanipulation with an absence of growth after micromanipulation. Then, a three modalities ordinal logistic regression model (absence of growth, growth of one colony, growth of more than two colonies) was applied to the data to evaluate the occurrence of each modality. Both proportional tests were performed using the R software (free license, version 3.4.3).

Finally, in a third step, the dataset was analyzed to compare the type of bacteria (Gram positive versus Gram negative), the respiratory type (aerobic versus anaerobic) and the type of microorganisms (bacteria versus yeasts) to evaluate the influence of each parameter on the growth after micromanipulation. As previously done, a Fisher exact test was applied to the dataset for each comparison using the R software (free license, version 3.4.3).

### Detection of a single cell of *S*. *enterica* in food samples

Following the ISO 6579 method, we used minced beef samples as matrices for testing the detection of a single cell of *Salmonella enterica subsp*. *enterica*. A single cell of *S*. *enterica* was inoculated into 225 ml of buffered peptone water (Merck ref. 1072208) and added to 25 g of minced beef in a stomacher bag (Gosselin, ref. BBAG-03). Bags were mixed for 30 seconds at 230 rpm on a stomacher system (Seward Stomacher® 400 Circulator) and incubated for 18h at 37°C. Then, 100 μl of the mixture were transferred into 10 ml tubes of Rappaport-Vassiliadis Salmonella (RVS) broth (Merck, ref. 146694) and incubated for 24h at 41.5°C. After incubation, xylose lysine deoxycholate (XLD) agar plates (Merck, ref. 146073) were inoculated with a loop of RVS broth and incubated for 24h at 37°C. Typical colonies of *S*. *enterica* were recorded and subcultured on TSA plates (Merck, ref. 146004) for identification, using a MicroSEQ 500 16S rDNA sequencing kit and system (Thermofisher Scientific, ref. 4346480 and ref. 4363198).

## Results

### Container for micromanipulation

In order to manipulate single microorganism cells, the cell suspension must be poured into a container allowing clear observation of the microorganism and easy movement of the capillary in three dimensions (X, Y, and Z axis). In addition, the container should not allow the non-specific binding of microorganisms on its surface.

Using these criteria, we compared six containers: the results are presented in Table 1.

**Table 1:** Comparison of containers, using three criteria.

Criterion	Container	Slide	33 mm plate	60 mm plate	90 mm plate	6-well plate
**Observation of cells**		+	++	++	-	-
**Capillary mobility**		++	-	++	+	-
**No cell binding**		-	+	+	+	+

(-) indicates that the criterion was not fulfilled, (+) that it was partially fulfilled, and (++) that it was completely fulfilled.

The 60 mm Petri dish offered the best combination of observation, capillary mobility, and non-binding surface.

### Capillary type

Three types of glass capillary were evaluated for the procedure, again taking three criteria:

- Capillary diameter: the capillary should not be clogged by microorganisms during sucking, especially by yeasts that might be around 7 μm in diameter.
- Length of the tip: this length must allow a good pressure balance between oil, air, and diluent inside the microcapillary, to avoid the uncontrolled sucking of bacterial suspension through capillarity
- Observation of the tip: clear observation of the tip through the microscope, without light diffraction, is essential to see cells being sucked into the microcapillary

As Table 2 shows, the TransferTip ES capillary was most suited to our application, thanks to its 15 μm diameter and 1 mm long tip. There was no risk of clogging while the microorganisms were being sucked up and the pressure balance between fluids was easy to obtain. In addition, its beveled design allowed us to observe the cells clearly during micromanipulations.

**Table 2:** Comparison of the microcapillaries, using three criteria.

Criterion	Type of capillary	IMSI/TESE	Polar Body Biopsy	TransferTip ES
**Tip diameter**		8 μm	20 μm	15 μm
		-	+	+
**Tip length**		1.9 mm	0.5 mm	1 mm
		-	-	+
**Design**		Straight, rounded	Straight, indented	Beveled tip
		-	+	+

(-) indicates that the criterion was not fulfilled, (+) that it was fulfilled.

### Culture method after micromanipulation

In order to determine whether the micromanipulation system could isolate one single viable bacterial cell, we had to develop a compatible culture protocol.

**Fig 1:**
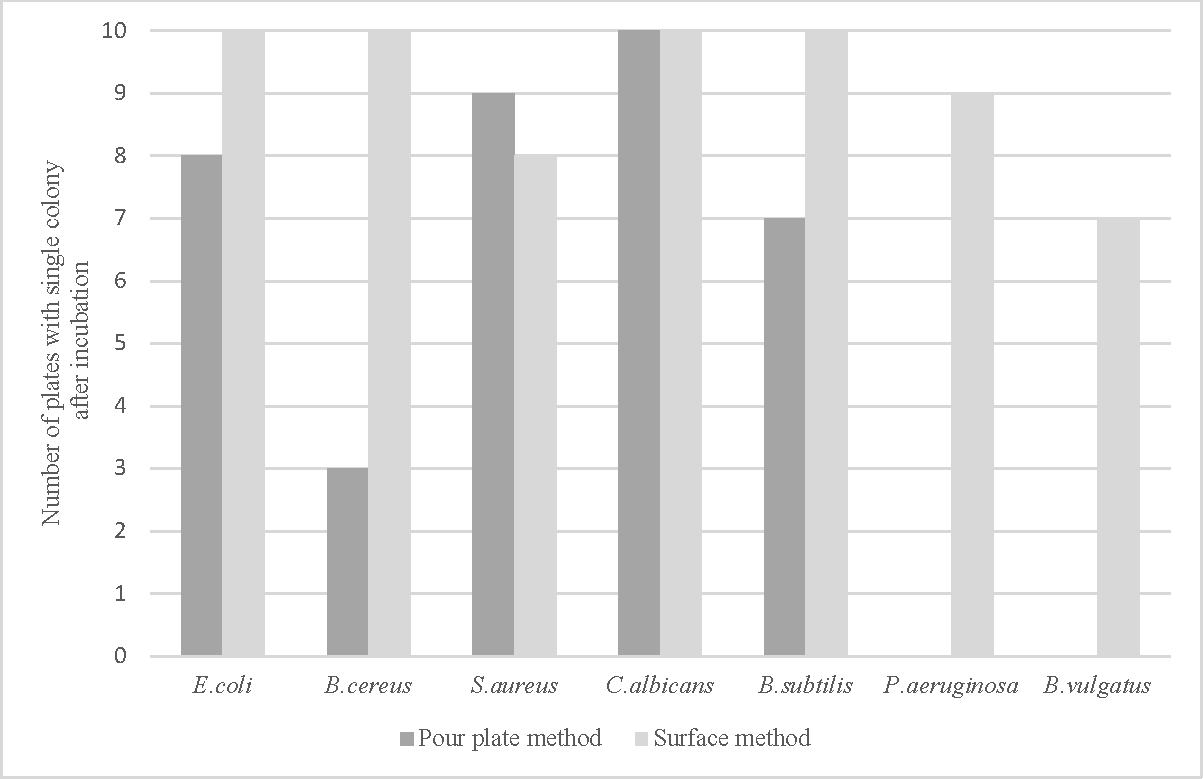
Growth of seven microorganisms according to seeding method after micromanipulation. The results are the number of plates showing a single colony after incubation (10 replicates per strain).

The first seeding method was based on a standard pour plate method with release of the single cell in 500 μl of physiological saline prior to the addition of culture medium. The second seeding method consisted in the release of the cell in a drop of diluent at the surface of a culture plate. Seven microorganisms were used to compare the two methods and the results are shown in Fig 1.

The release of single cell in a drop at the surface of an agar plate showed the best levels of growth for aerobic bacteria, and it was the only method that allowed the growth of *P*.*aeruginosa*. For the anaerobic bacterium *B*.*vulgatus*, only releasing a cell at the surface of a COS agar plate showed any colony formation after incubation. The impossibility of adding blood to the Schaedler medium used for the pour plate method had probably resulted in an inadequate nutrient medium. Lastly, the two methods showed similar results for the yeast *C*.*albicans*.

### Determination of the diluent

The diluent used for the microbial suspension could have a negative impact on single cell manipulation if a high concentration of particles is present or if the color disturbs the observation under the microscope. In addition, the diluent should keep the microorganisms in a viable state without impacting their subsequent growth.

Based on the criteria of transparency and absence of particles, physiological saline and R2B medium gave the best microscopic observations.

**Fig 2:**
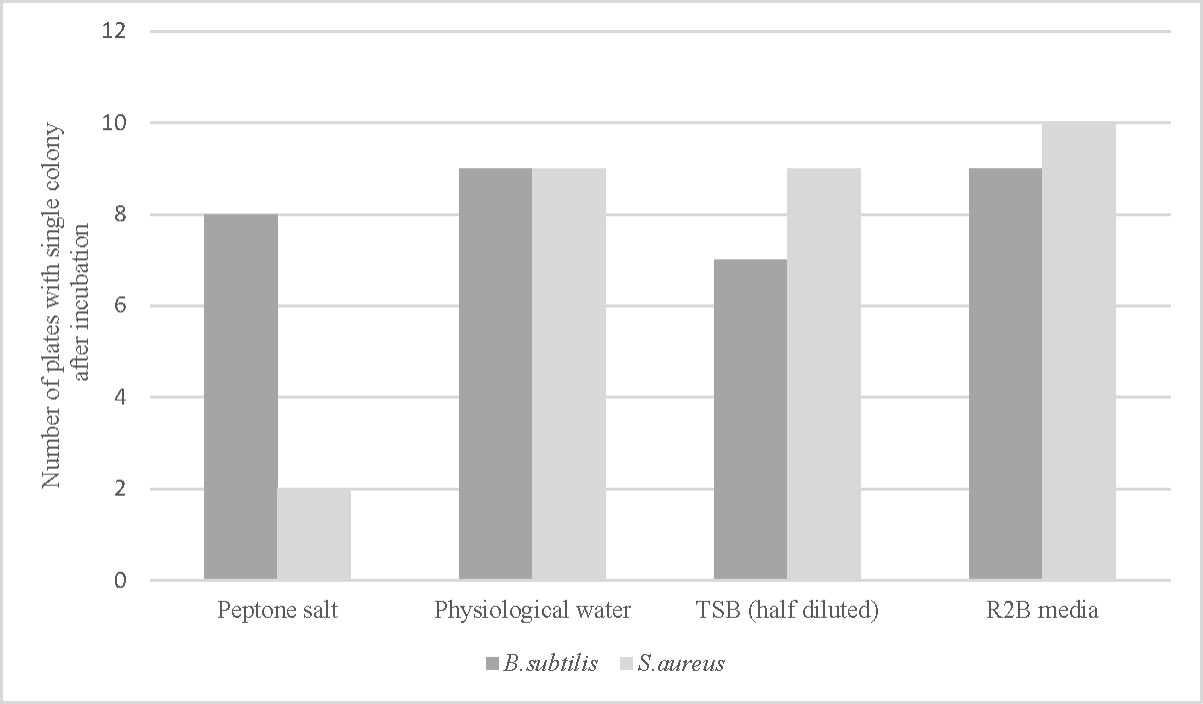
Growth of *B*.*subtilis* and *S*.*aureus* according to diluent after micromanipulation. The results are the number of plates showing a single colony after incubation (10 replicates per strain).

The number of plates with a single colony after incubation was recorded for the two test bacteria (*B*.*subtilis* and *S*.*aureus*), as shown in Fig 2. When peptone salt was used as the diluent, growth rates of 80% and 20% were obtained for *B*.*subtilis* and *S*.*aureus*, respectively. For half-diluted TSB, the growth rates were 70% for *B*.*subtilis* and 90% for *S*.*aureus*. The highest growth rates after micromanipulation were observed for physiological saline and R2B media, giving 90% and 90–100% of single colony growth in both cases.

As R2B and physiological saline gave similar results, the saline solution was selected as the main diluent for the protocol of the subsequent experiments.

### Effect of the physiological state of the bacteria on micromanipulation

Earlier work has shown that the growth rate after micromanipulation is dependent on the growth phase and physiological state of the microorganism [8]. *E*.*coli* (Gram-negative rods) and *K*.*rhizophila* (Gram-positive cocci) were used as models to study the impact of the physiological state on the growth rate after single cell manipulation.

**Fig 3:**
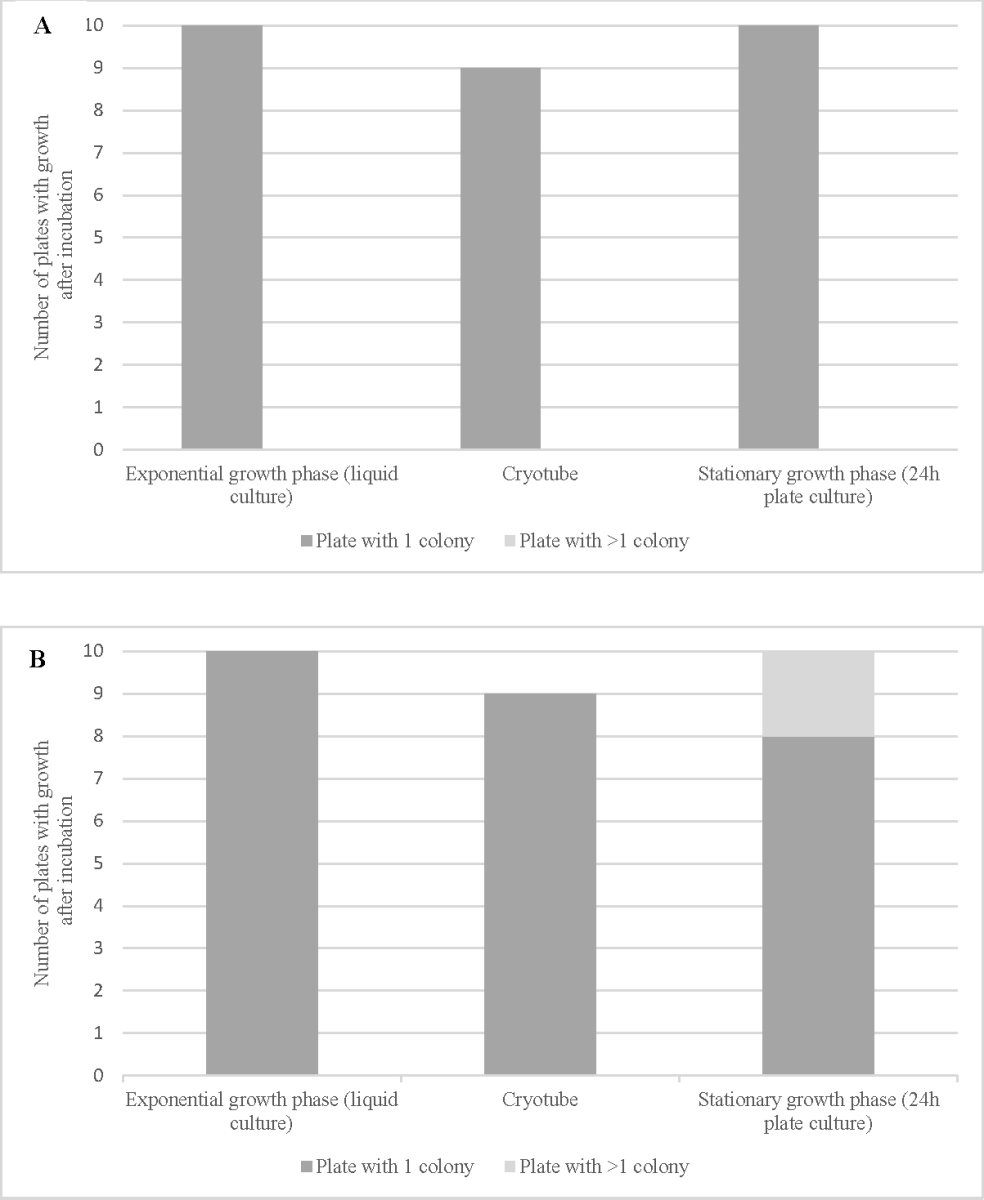
**Growth rate after micromanipulation for single cells of *E*.*coli* (A) and *K*.*rhizophila* (B) in various physiological states.** The results are the number of plates showing one single colony or more after incubation (10 replicates per strain).

Results presented in Fig 3 show similar growth rates for the three starting conditions tested. The stationary growth phase from 24h cultures was selected as the standard culture condition.

### Evaluation of the growth rate after micromanipulation

Following the method described previously, 13 strains of a wide range of microorganisms were tested for growth after micromanipulation. Ten replicates were performed per strain.

**Fig 4:**
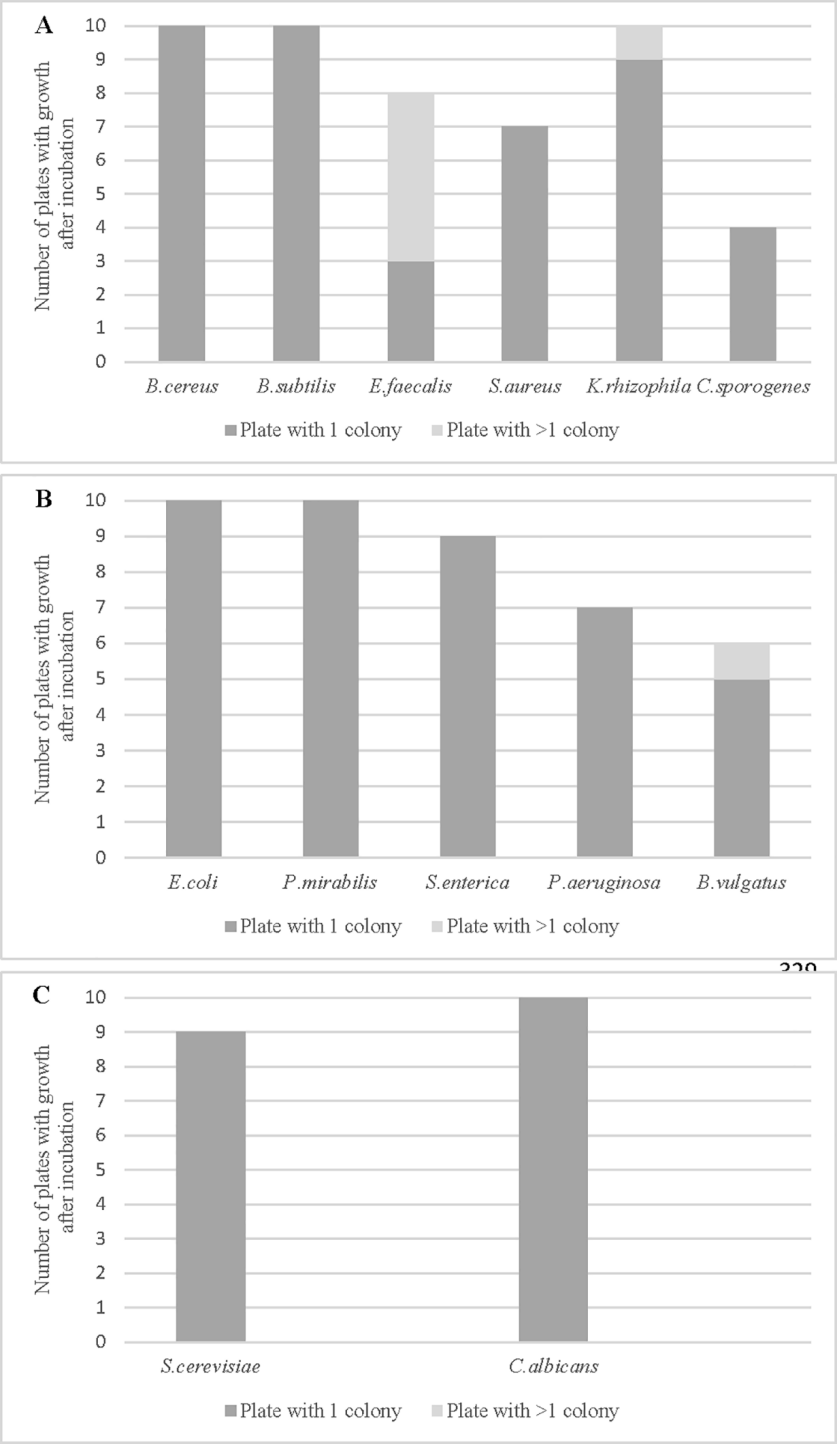
**Number of plates with growth after micromanipulation of (A) Gram-positive bacteria, (B) Gram-negative bacteria and (C) yeasts.** The results are the number of plates showing one colony or more after incubation (10 replicates per strain).

As shown in Fig 4A below, three strains of Gram-positive bacteria (*B*.*cereus*, *B*.*subtilis*, and *K*.*rhizophila*) demonstrated single cell growth rate of 100% after micromanipulation. *E*.*faecalis* and *S*.*aureus* showed 70–80% growth rate, although five plates with *E*.*faecalis* presented growth of more than one colony. For Gram-negative bacteria (Fig 4B), both *E*.*coli* and *P*.*mirabilis* showed 100% growth rate after micromanipulation. *S*.*enterica* and *P*.*aeruginosa* showed growth rates of 90% and 70%, respectively. For anaerobic bacteria, the growth rate was slightly less, with *B*.*vulgatus* showing 60% growth and *C*.*sporogenes* 40% (Fig 4A and 4B). Finally, the yeasts *S*.*cerevisiae* and *C*.*albicans* demonstrated single cell growth rates of 90% and 100%, respectively (Fig 4C).

To highlight the ability of cells to growth after micromanipulation, a statistical analysis has been used. With a true probability of success greater than 0.5, the Fisher-exact test demonstrated a 95% confidence interval to observe a growth of cells after micromanipulation. Then, to demonstrate the effectiveness of the micromanipulation method to obtain one colony from one single cell, we performed an ordinal logistic regression model on the whole dataset. Whatever the bacteria considered from the 13 strains tested, the appraisal from the statistical analysis showed a probability to have one single colony of 79.2%, 15.4% to have no growth and 5.4% to have more than one colony after micromanipulation.

Analyzing further the results, a statistical analysis has been used to evaluate the influence of the type of bacteria, the respiratory type or the type of microorganisms on the cells’ growth after micromanipulation. When comparing results from Gram positive and Gram negative bacteria, the Fisher-exact test with a P-value > 0.05 demonstrated no significant difference in growth of cells after micromanipulation between both types of bacteria. Same result was demonstrated when comparing dataset from bacteria and yeasts. However, when comparing results from aerobic and anaerobic bacteria, with a P-value < 0.05 the Fisher-exact test demonstrated a significant statistical difference between both respiratory types.

These results showed that most of the microbial cells were able to grow on culture plates after the micromanipulation and isolation of a single cell using the new test method. Nevertheless, the respiratory type of bacteria has an influence on the growth rate after micromanipulation.

### Detection of a single cell of *S*. *enterica* in food samples

The micromanipulator system and method was used to evaluate the detection of a single cell of *S*. *enterica* in minced beef. The objective of this test was to verify that the ISO 6579 method can detect one cell of *S*. *enterica* in 25 g of meat.

As shown in Table 3, six out of the ten meat samples showed typical colonies of *S*. *enterica* growing on XLD agar plates (red colonies with black centers) after single cell inoculation. To confirm the results, a typical colony from each sample was subcultured and sequenced. Sequencing confirmed *Salmonella enterica* in the colonies tested from positive samples and thereby confirmed that the ISO method can detect single cells of *S*. *enterica* in meat samples.

**Table 3:** Growth of typical colonies of *S*.*enterica* in food samples and results of sequencing.

Replicates	Typical colonies of *Salmonella*	Identification
**Negative control**	-	N/A
**Sample 1**	-	N/A
**Sample 2**	-	N/A
**Sample 3**	+	*S*.*enterica subsp*. *enterica*
**Sample 4**	-	N/A
**Sample 5**	+	*S*.*enterica subsp*. *enterica*
**Sample 6**	-	N/A
**Sample 7**	+	*S*.*enterica subsp*. *enterica*
**Sample 8**	+	*S*.*enterica subsp*. *enterica*
**Sample 9**	+	*S*.*enterica subsp*. *enterica*
**Sample 10**	+	*S*.*enterica subsp*. *enterica*

## Discussion

Since the introduction of micromanipulation techniques for the isolation of single cells from environmental samples 40 years ago, several attempts have been made to improve the micromanipulation of single microbial cells [9, 11, 13]. Each time, suggestions were based on the state of the art at that time. With further improvements in microscopy and the development of hydraulic systems such as CellTram, the capillary can be positioned quickly and precisely, which increases the efficiency of micromanipulation of such small cells as microorganisms.

According to other studies [10, 11], the capillary used for micromanipulation of bacterial cells must have a beveled tip to allow clear observation of cells during sucking, and a diameter greater than 10 μm to avoid clogging. On the basis of its 15 μm diameter and beveled tip, we selected the TransferTip (ES) capillary for developing our method.

Previous work has shown limited recovery rates in culture media when single cells were isolated after being spread on a microscope slide [9]. To improve the growth rate by avoiding cell drying and desiccation on a slide during the micromanipulation, we decided to work with microbial cells resuspended in 6 ml of an isotonic solution (0.9% saline). Moreover, this diluent was clear and free of particles, allowing us to see the cells clearly. This is particularly important when manipulating cocci.

As the objective of our study was to determine whether viable single microbial cells could be isolated using a micromanipulator, it was essential for us to establish the optimal method for culturing these cells. We compared pour plate and spread plate methods, releasing the cell in a drop of isotonic diluent. The results presented in Fig 1 show a correlation between the bacterial respiratory type (aerobes, facultative anaerobes) and growth on media. Isolated cells from facultative anaerobes such as *E*.*coli*, *S*.*aureus* and *B*.*cereus* grew with both seeding methods, although the spread plate technique gave higher recovery rates for *E*.*coli* and *B*.*cereus*. On the other hand, single cells of the strictly aerobic *P*.*aeruginosa* did not grow when the pour plate method was used but showed a 70% growth rate with the spread plate method. As mentioned in an earlier study [14], the growth rate of obligate aerobes is reduced when using the pour plate method and the risk of killing heat-sensitive cells is higher with hot agar. In addition, it was not possible to add blood to the culture media for the anaerobic bacterium *B*.*vulgatus* with the pour plate method, resulting in the absence of colony growth.

To determine the optimal physiological state for the microorganisms to survive micromanipulation, we evaluated the growth rate after isolating single cells from exponential and stationary growth cultures. Previous work [8] showed growth rates after the micromanipulation of bacteria isolated from exponential phase cultures to be slightly higher than those from the stationary phase, corresponding to the ratio of live/dead cells in the initial source cultures. In our study, tests demonstrated that the initial physiological state had no influence on colony formation after single cell isolation for either rods or cocci. Indeed, using our newly developed micromanipulation method, the operator was able to select a higher ratio of viable *E*.*coli* cells by taking visibly swimming bacteria from the 6 ml isotonic solution. Earlier studies had no possibility of discriminating living from dead cells [8].

**Fig 5:**
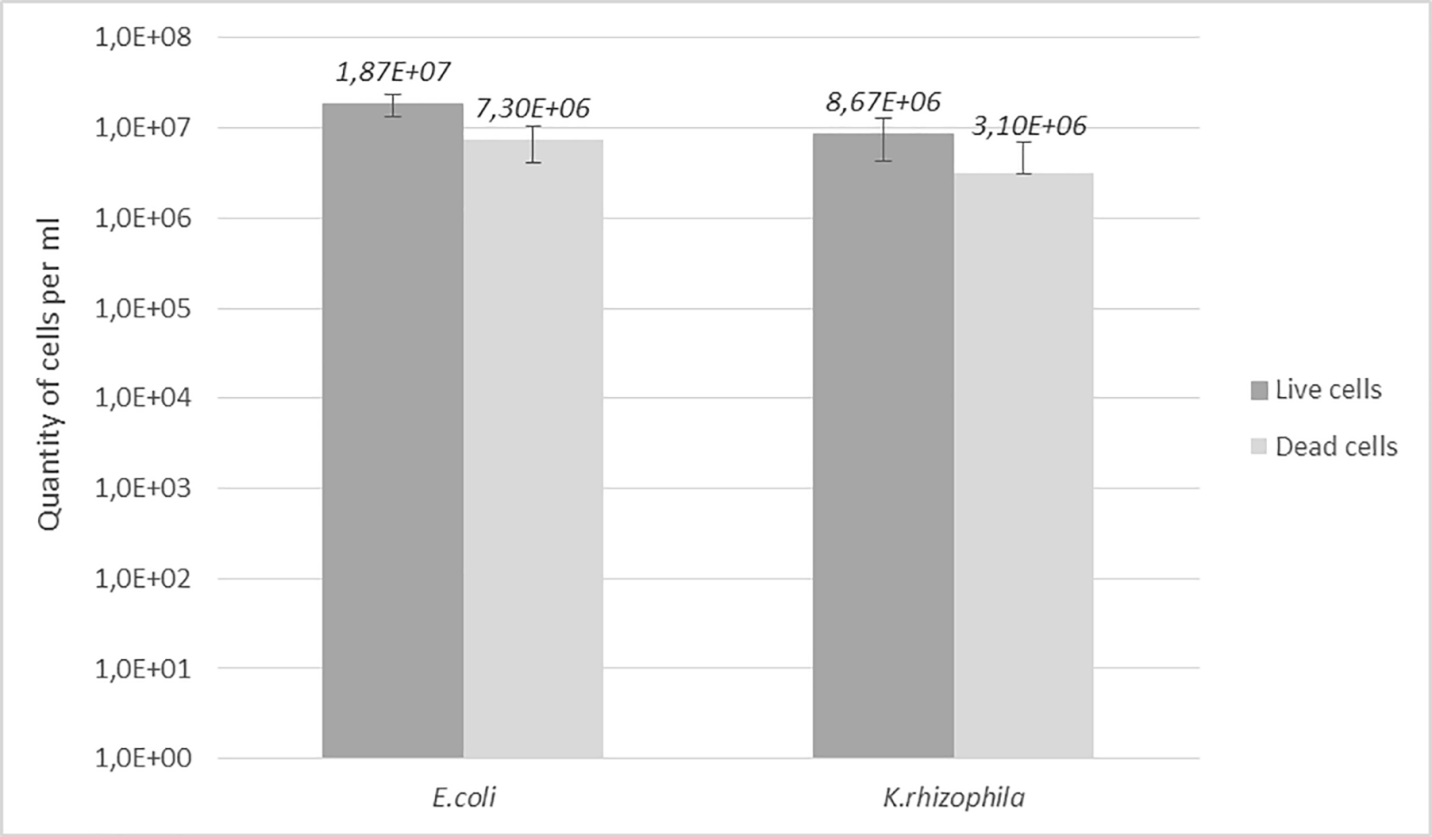
Viable and dead cells of *E*.*coli* and *K*.*rhizophila* after freezing in cryotubes and thawing at room temperature. Each result is expressed as a mean of six measurements performed on a Guava flow cytometer after labelling the bacterial cells with CFDA and PI.

We also evaluated the effect of cold stress on bacterial cells before micromanipulation. Stationary phase cultures of *E*.*coli* and *K*.*rhizophila* were frozen at -80°C in cryotubes before being thawed at room temperature immediately before micromanipulation. Results showed no difference in the growth rate of single cells for bacteria coming from 24h culture plates or from cryotubes. We performed a complementary study using flow cytometry to evaluate the proportion of viable and dead cells after freezing. Bacterial cells were labelled using 5(6)-carboxyfluorescein diacetate (CFDA) at a final concentration of 23 μg/ml (Sigma ref. 21879) and propidium iodide (PI) at a final concentration of 20 μg/ml (Sigma ref. P4864) to differentiate viable and dead cells. The labelled cells were then analyzed using a Guava flow cytometer (Merck ref. 0500–4005). The viable cell concentrations in the thawed cryotubes were 72% for *E*.*coli* and 74% for *K*.*rhizophila*, as shown in Fig 5. The high numbers of viable cells after freezing and, in the case of *E*.*coli*, the possibility for the operator to select motile cells (assumed to be viable) visually, increased the chances of successful growth from a mixed population of dead, stressed, and viable bacteria after a freeze/thaw cycle.

In our study, 13 microorganisms representing a wide range of physiology and structure were evaluated for growth after single cell micromanipulation. Statistical analysis of the dataset demonstrated an overall total successful growth rate confidence interval of 95% for the developed method. Considering only the replicates with a single colony, a statistical test based on ordinal logistic regression model estimated the growth probability of the method to be 79.2%. In detail, Gram-positive bacteria, Gram-negative bacteria and yeasts showed single colony growth rates of 71.7%, 82% and 95%, respectively. Because cocci are small in size and have a tendency to stick together (in particular *E*.*faecalis*, arranged in short chains), the isolation of single bacteria was less optimal than for rods or yeasts and could explain why Gram-positive bacteria gave the lowest precision. However, a deeper statistical analysis using an exact proportional test demonstrated no significant difference in growth success when comparing together Gram-positive and Gram-negative bacteria or bacteria and yeasts.

Nevertheless, a statistically significant difference was observed between aerobic and anaerobic bacteria. Even if the number of tested anaerobic bacteria was limited (only two bacteria in our study), improvements of the micromanipulation method of anaerobic bacteria would have to be considered to increase the growth efficiency of this bacterial respiratory type.

The aim of this study was to develop and evaluate a micromanipulation method to challenge the LOD ability of standards and rapid pathogen detection methods. Previous work [8] and our study demonstrate that single cells of *S*.*enterica* can grow when inoculated directly onto culture media. The results of food samples presented in Table 3 show the successful detection of single cells of *S*.*enterica* in 25 g of minced beef, even though the growth rate of 60% was slightly lower than with the direct inoculation of isolated bacterial cells onto culture plates (Fig 4B). One hypothesis to explain this lower figure is that the meat’s natural flora could interfere with the growth of *Salmonella* cells. In addition, the successive steps and dilutions of the reference method (only 100 μl of 225 ml pre-enrichment broth were transferred to RVS broth tubes) could impact the result if only low levels or no *Salmonella* at all are transferred.

In conclusion, the method we present in this study demonstrates the successful handling of a wide variety of single isolated microbial cells, with high subsequent growth rates. In addition, we have highlighted the current reference method’s ability to detect low levels of *Salmonella* in food samples, confirming the efficient detection of single *Salmonella* cells in meat. Other applications, especially sterility tests for pharmaceutical products, would also benefit from this new method of single cell manipulation.

## Supporting information

S1 File Compilation of raw data. The file below contains all the data from each growth rate studies.
(XLSX)
